# Increase of SARS-CoV-2 RNA load in faecal samples prompts for rethinking of SARS-CoV-2 biology and COVID-19 epidemiology

**DOI:** 10.12688/f1000research.52540.3

**Published:** 2021-07-01

**Authors:** Mauro Petrillo, Carlo Brogna, Simone Cristoni, Maddalena Querci, Ornella Piazza, Guy Van den Eede

**Affiliations:** 1 European Commission, Joint Research Centre (JRC), Ispra, Italy; 2 Craniomed group srl, Montemiletto, Italy; 3 ISB Ion Source & Biotechnologies Srl, Bresso, Italy; 4 Department of Medicine and Surgery, University of Salerno, Baronissi, Italy; 5 European Commission, Joint Research Centre (JRC), Geel, Belgium

**Keywords:** SARS-CoV-2, COVID-19, gut microbiota

## Abstract

Background

Scientific evidence for the involvement of human microbiota in the development of COVID-19 disease has been reported recently. SARS-CoV-2 RNA presence in human faecal samples and SARS-CoV-2 activity in faeces from COVID-19 patients have been observed.

Methods

Starting from these observations, an experimental design was developed to cultivate *in vitro* faecal microbiota from infected individuals, to monitor the presence of SARS-CoV-2, and to collect data on the relationship between faecal bacteria and the virus.

Results

Our results indicate that SARS-CoV-2 replicates *in vitro* in bacterial growth medium, that the viral replication follows bacterial growth and it is influenced by the administration of specific antibiotics. SARS-CoV-2-related peptides have been detected in 30-day bacterial cultures and characterised.

Discussion

Our observations are compatible with a ‘bacteriophage-like’ behaviour of SARS-CoV-2, which, to our knowledge has not been observed or described before. These results are unexpected and hint towards a novel hypothesis on the biology of SARS-CoV-2 and on the COVID-19 epidemiology. The discovery of possible new modes of action of SARS-CoV-2 has far-reaching implications for the prevention and the treatment of the disease.

## Introduction

Recent scientific articles and reviews ^1– 3^ discuss the relationship between gastrointestinal microbiota and COVID-19 disease. In particular, the prolonged presence of SARS-CoV-2 RNA in human faecal samples from COVID-19 patients has been reported ^4^ and the potential role of orofecal transmission of SARS-CoV-2 has been examined in systematic reviews ^5, 6^ and open evidence briefs ^7, 8^. Cases of SARS-CoV-2 detection in faecal samples from patient with typical symptoms of COVID-19 but negative to multiple SARS-CoV-2 real-time reverse transcription polymerase chain reaction (rRT-PCR) tests on oropharyngeal and nasopharyngeal swabs have been reported ^9^. SARS-CoV-2 faecal viral activity was depicted in association with gut microbiota composition in patients with COVID-19 ^10^, and the replicating virus was detected in faeces ^11^. At the same time, Wölfel *et al*. ^12^ observed high viral RNA concentration in stool samples, but reported isolation of infectious virus only from throat- and lung-derived samples, while Yao *et al*. ^13^ had indication of viable SARS-CoV-2 particles in stool samples, denoting that the detailed biology of SARS-CoV-2 is not yet fully elucidated. Moreover, the interaction between SARS-CoV-2 and individual variable microbiota composition could drive the differential pathophysiological effects and severity of symptoms (Yeoh YH *et al*. 2021, Zuo T *et al*. 2021, Zuo T *et al*. 2020).

Our experiments further explored the relationship between COVID-19 disease and SARS-CoV-2 infected faeces to provide data relevant for pandemic understanding and disease management. The results however did not correspond with current thinking of the epidemiology of SARS-CoV-2 and, therefore, we believe a quick sharing with the scientific community of our findings is imperative.

## Methods

The experimental design included:

1) the inoculation of NutriSelect™ Plus nutrient broth at 37°C, fit for the growth of more fastidious bacteria, with a faecal sample (stool) from one subject positive to SARS-CoV-2 and from a healthy individual (here called sample A and sample B, respectively) following written informed consent.
2) The assessment of the presence of SARS-CoV-2 RNA in both samples, after seven days of culture, using the Luminex technology; with confirmation of the presence of SARS-CoV-2 RNA in sample A, and of its absence in sample B.
3) Inoculation of sample B with the supernatant of sample A, obtained after centrifugation (hereafter called sample B _(A+)_) and resuspension of the formed pellet (sample C).
4) Incubation of all samples (A, B, B _(A+)_ and C) for 30 days under the same conditions in NutriSelect™ Plus nutrient broth at 37°C with measurement of the viral RNA load in each sample at days 1, 2, 3, 7, 14, 21, and 30, following the date of inoculation (day 0).
5) Antibiotic treatment on 18 aliquots derived from sample B _(A+)_ at day 21, consisting in the addition of a specific antibiotic (each of the following: metronidazole, clindamycin, lincomycin, piperacillin+tazobactam, vancomycin, amoxicillin, ampicillin, cefixime, ceftriaxone, meropenem, rifaximin, azithromycin, erythromycin, gentamicin, ciprofloxacin, colistin, levofloxacin, and teicoplanin) to each aliquot. SARS-CoV-2 RNA load was measured by Luminex technology in each aliquot before and 3 days after antibiotic administration.
6) In parallel, additional analyses were performed to evaluate and monitor over time the bacterial growth and metabolic activity of all samples and all aliquots of sample B _(A+)_, using SANIST Biotyper according to the method described by Cristoni *et al.* ^14^
7) Purification and analysis of the peptides present in sample B _(A+)_ at day 30.

The details of the procedures and protocols used are presented in the extended data, together with a schematic representation of the experimental design (Graphical abstract) ^15^.

## Results

The experimental design included a series of analyses (performed on all samples A, B, B _(A+)_ and C) aimed at verifying: 1) the permanence/survival over time and the eventual multiplication of SARS-CoV-2 RNA *in vitro*; 2) the presence/synthesis of SARS-CoV-2 peptides in the cultures having confirmed SARS-CoV-2 RNA presence; 3) the effect of antibiotics administration in sample B _(A+)_; 4) the concomitant presence of other metabolites; and 5) the characterisation of the bacterial samples, including the verification of the presence of eukaryotic cells.

### Presence of SARS-CoV-2 RNA

**Figure 1.**
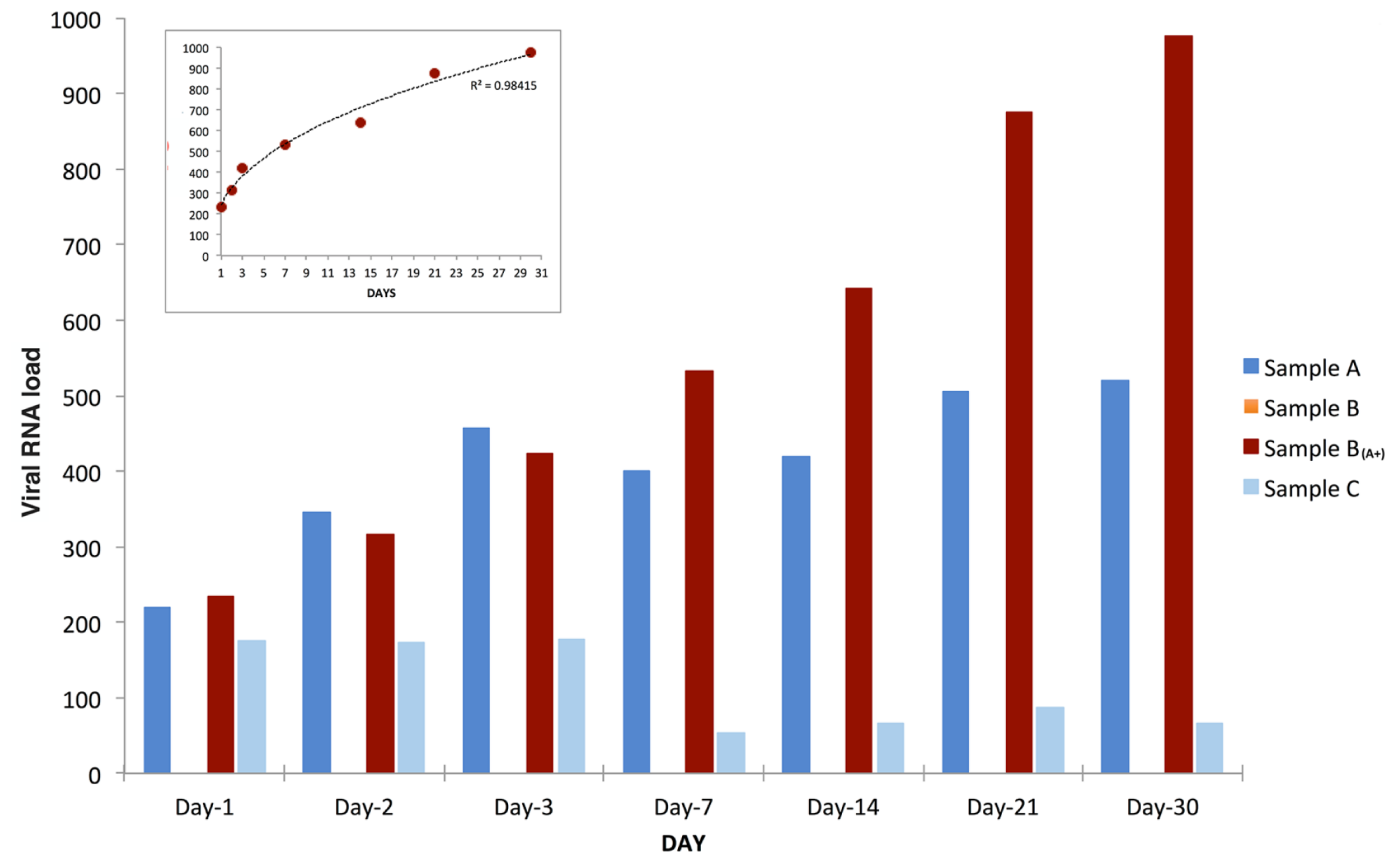
SARS-CoV-2 RNA load variation over time. SARS-CoV-2 RNA load measurements (reported as AU, see extended data) of samples A (blue bars), B (orange bars), B _(A+)_ (red bars), and C (azure bars) grown, all under the same conditions for thirty days from inoculation (day 0). SARS-CoV-2 RNA load in sample B _(A+)_ had a power increase trend over time (as shown in the small frame on top-left), slightly increased in sample A, and decreased in sample C. As expected, sample B was found constantly negative.

The results presented and discussed here, carried out over a period of 30 days, confirmed the extra-corporal multiplication of SARS-CoV-2 RNA: viral load highly increased over time in sample B _(A+)_, slightly increased in sample A, decreased in sample C while, as expected, sample B was found constantly negative ( Figure 1).

**Figure 2.**
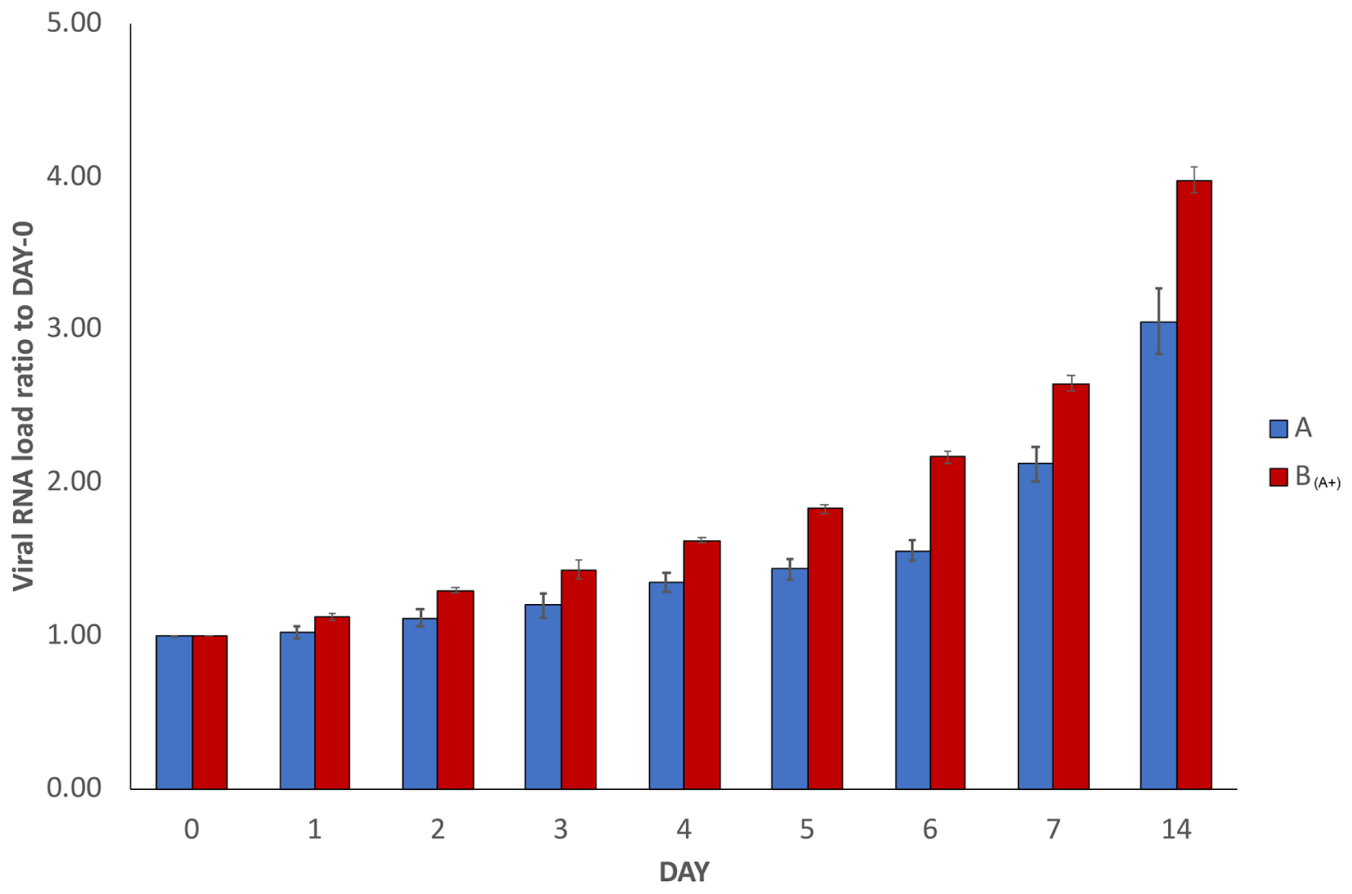
Average SARS-CoV-2 RNA load variation in repetitions. The graph reports average results of three repetitions of the experiment conducted using the same starting material as described in Figure 1 (with the exception that the repetitions were stopped at day 14 instead of day 30). To normalise the measurements, all values at day 0 were used as denominator (at day 0 all values = 1), *i.e.* for each sample, at day X, the ratio between LuminexCountAtDayX/LuminexCountAtDay0 was calculated. Each bar represents the average of the SARS-CoV-2 RNA load ratio of samples A (blue bars) and B _(A+)_ (red bars), together with the calculated standard deviations.

In order to verify the reproducibility of our results, the whole experiment was repeated independently three times using the same infected and healthy samples (with the exception that the repetition experiments were stopped at day 14 instead of day 30). The results of the SARS-CoV-2 RNA load measurements in the repetitions are reported in Figure 2, where results are depicted as the average of the measurements of the three repetitions, together with the calculated standard deviations. The trend was confirmed, with the increase over time of measured viral RNA load in sample A and sample B _(A+)_. Decrease in sample C and no detection in sample B were also confirmed, but they are not reported in Figure 2.

**Figure 3.**
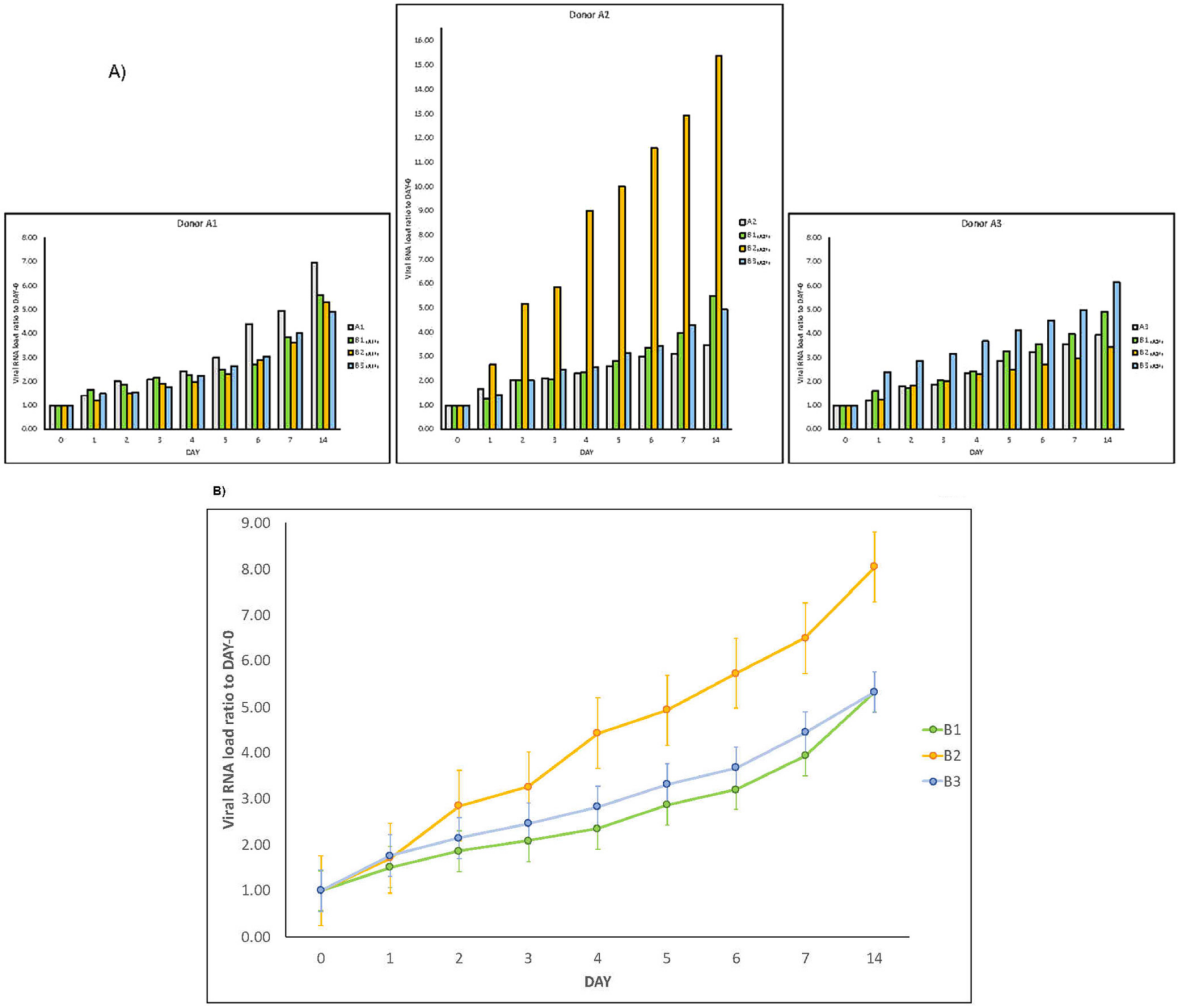
SARS-CoV-2 RNA load increase in different donor and recipient samples. Results of experiments combining samples from three “infected donor” sources (A1, A2 and A3) and from three “healthy recipient” sources (B1, B2 and B3). **A**) The graphs report results of nine combinations. To normalise the measurements, all values at day 0 were used as denominator (at day 0 all values = 1), i.e. for each sample, at day X, the ratio between LuminexCountAtDayX/LuminexCountAtDay0 was calculated. Each bar represents the SARS-CoV-2 RNA load ratio. Although with certain differences, the observed trends are similar, confirming the increase over time of SARS-CoV-2 RNA load in samples of type A and samples of type B _(A+)_, independently from the sources of A and B. **B**) Each line represents the average of the SARS-CoV-2 RNA load ratio of samples B1 (green line), B2 (yellow line), and B3 (azure line) infected each one with three different A donor sources. To normalise the measurements, all values at day 0 were normalised as described in panel A).

In addition, three new couples of faecal samples from different “infected donors” ( *i.e.* sources of A) and “healthy recipients” (i.e. sources of B) have been recruited, and subject to the same experimental procedure. Samples were collected from anonymous donors, and no information ( *i.e.* age, sex, blood serotype, severity of the disease, time of the collection, fatality, *etc.*) is available. All combinations of “infected donors” sources (A1, A2 and A3) and “healthy recipients” donors (B1, B2 and B3) were subject to the same experimental procedure. Although with certain differences, the observed trends are similar ( Figure 3A), confirming the increase over time of SARS-CoV-2 RNA load in samples of type A and in samples of type B _(A+)_ . Lastly, for each “recipient”, we measured SARS-CoV-2 RNA load ( Figure 3B).

### Effect of antibiotics administration

**Figure 4.**
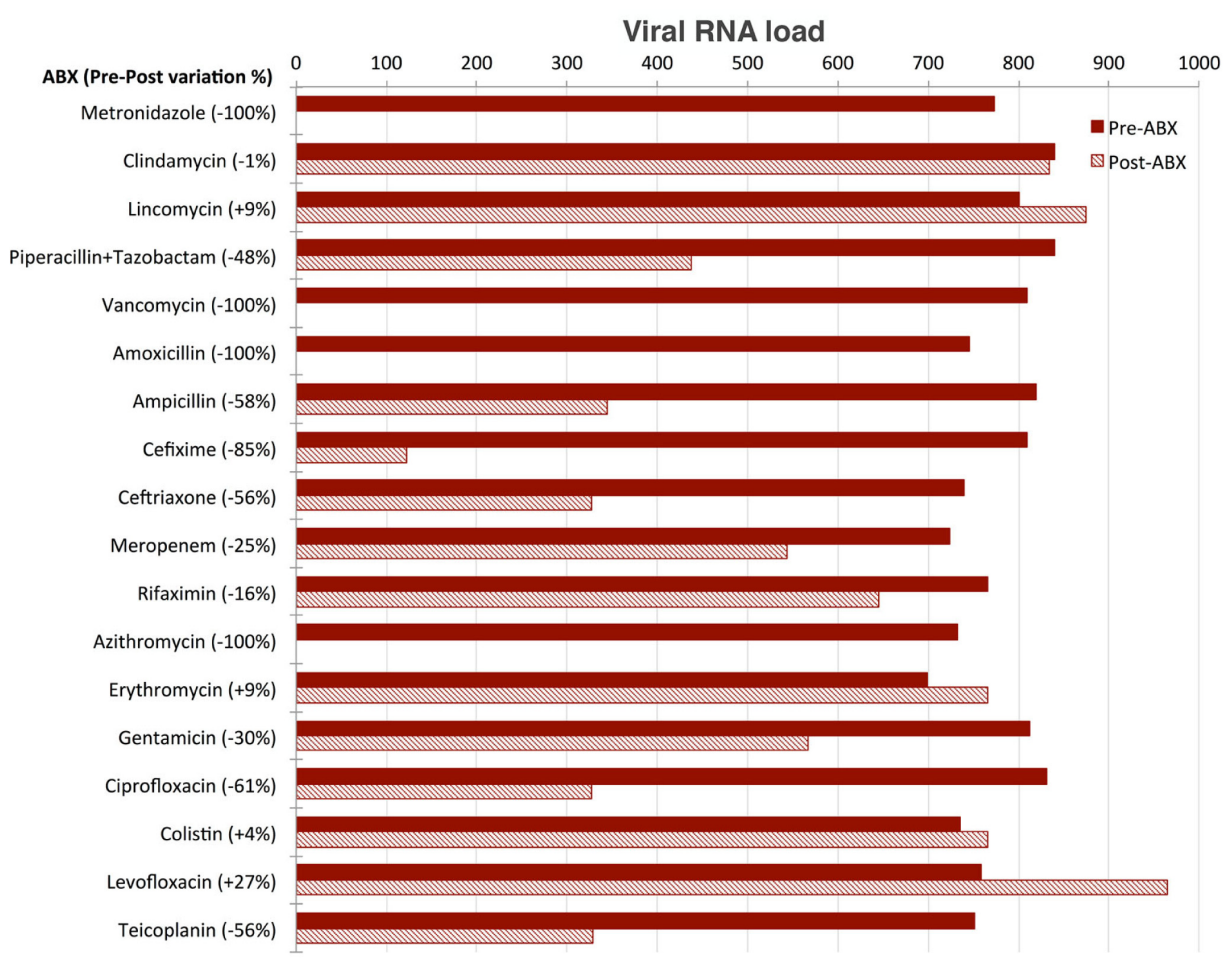
Effect of antibiotics on viral load. SARS-CoV-2 RNA load measurements (reported as AU, see Supplementary Material) of eighteen aliquots pre- (red) and post- (three days, dashed) treatment with the following selection of antibiotics (ABX): Metronidazole (class: Azoles); Clindamycin, Lincomycin, Piperacillin+Tazobactam, Vancomycin (class: Carboxylic acids and derivatives); Amoxicillin, Ampicillin, Cefixime, Ceftriaxone, Meropenem (class: Lactams); Rifaximin (class: Macrolactams); Azithromycin, Erythromycin, Gentamicin (class: Organooxygen compounds); Ciprofloxacin, Colistin, Levofloxacin (class: Quinolines and derivatives); Teicoplanin (semisynthetic glycopeptide antibiotic). SARS-CoV-2 RNA load is reported as preABX-postABX variation in percentage.

Aliquots of sample B _(A+)_ tested after three days of culture in the presence of the single different antibiotics belonging to different classes were analysed and the SARS-CoV-2 RNA load measured in each of them. SARS-CoV-2 RNA load was found to be influenced by the presence of antibiotics in different ways ( Figure 4):

- SARS-CoV-2 RNA load was reduced to undetectable levels in the four aliquots treated with metronidazole, vancomycin, amoxicillin and azithromycin, respectively.
- SARS-CoV-2 RNA load decreased by 20% to 85% in the aliquots treated with piperallicin+tazobactam, ampicillin, cefixime, ceftriaxone, meropenem, gentamicin, ciprofloxacin and teicoplanin. For example, cefixime induced a decrease of viral RNA load of 85%, ciprofloxacin of 61% and teicoplanin of 56%.
- SARS-CoV-2 RNA load was not substantially affected by the presence of clindamycin, lincomycin, rifaximin, erythromycin, colistin and levofloxacin.

### Presence of SARS-CoV-2 peptides

After 30 days of bacterial growth in culture, aliquots of samples B _(A+)_ (from couple 0) were collected and tested for the presence of SARS-CoV-2-related peptides using mass spectrometry (details are described in extended data ^15^). Several peptides found in the aliquot from sample B _(A+)_ were assigned to SARS-CoV-2 proteins. As shown in Table 1, the sequence of some of the peptides (pep51 and pep121, matching on NSP3; pep199, matching on the spike protein; pep25 and pep68, matching on NS3 and N, respectively) have one or more amino acid (AA) changes (highlighted in red) with respect to the translations of CDS regions reported in the reference ‘Severe acute respiratory syndrome coronavirus 2 isolate Wuhan-Hu-1, complete genome sequence’ (GenBank LOCUS: NC_045512.2). No SARS-CoV-2-related peptide was identified in the aliquot of sample B.

**Table 1.** Examples of 12 peptides (named as in the first column) mapping on different SARS-CoV-2 proteins (column “Match”) are here reported. Amino acids highlighted in red represent changes with respect to the translations of CDS regions reported in the reference severe acute respiratory syndrome coronavirus 2 isolate Wuhan-Hu-1, complete genome sequence (GenBank LOCUS: NC_045512.2). Pep51, pep121, and pep230 were found with a different mass spectrometry approach using the Q Exactive HF Hybrid Quadrupole-Orbitrap with an ultra-high-field analyser (Brogna, personal communication).

Peptide ID	Length (AA)	Fragment	Match	From	To
pep51	27	ESDDYI KLNGPL TVGGSC LLSGHNLAK	NSP3	268	294
pep121	82	L ILSVCLGSLIYSTAALGVLMSNLGMPSYCTGYREGYLNSTNVTIATYCTGSIPCSVCLSGLDSLDTYPSLETIQITISSFK		1416	1497
pep20	77	CLGSLIYSTAALGVLMSNLGMPSYCTGYREGYLNSTNVTIATYCTGSIPCSVCLSGLDSLDTYPSLETIQITISSFK		1421	1497
pep230	62	WVLNNDYYRSLPGVFCGVDAVNLLTNMFTPLIQPIGALDISASIVAGGIVAIVVTCLAYYFM	NSP4	241	302
pep199	62	TDAVRDPQTLEILDITPCSFGGVSVITPGTNTSNQVAVLYQ GVNCTEVPVAIHADQLTPTWR	S	573	634
pep33	69	DPQTLEILDITPCSFGGVSVITPGTNTSNQVAVLYQDVNCTEVPVAIHADQLTPTW SVYSTGSNVFQTR		578	646
pep190	74	SVASQSIIAYTM LLGAENSVAYSNNSIAIPTNFTISVTTEILPVSMTKTSVDCTMYICGDSTECSNLLLQYGSF		686	759
pep181	75	SIIAYTMSLGAENSVAYSNNSIAIPTNFTISVTTEILPVSMTKTSVDCTMYICGDSTECSNLLLQYGSFCTQLNR		691	765
pep103	31	IQDSLSSTASALGKLQDVVNQNAQALNTLVK		934	964
pep25	77	DCVVLHSYFTSDYYQLYSTQLSTDTGVEHVTFFIYNKIVDEPEEHVQIHTIDGSSGVVNPVMEPI CDEPTTTTSVPL	NS3	199	275
pep68	13	G ISP GRMAGNGGDAALALLLLDR	N	204	216
pep38	31	EAVGTNLPLQLGFSTGVNLVAVPTGYVDTPN	NSP14	99	129

**Figure 5.**
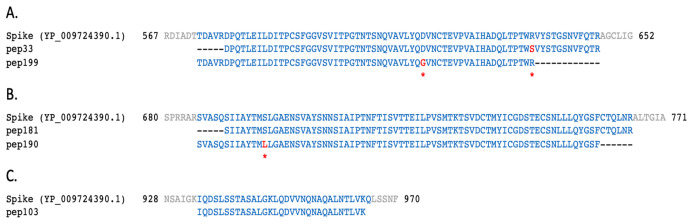
Peptides identified in sample B _(A+)_ mapped on SARS-CoV-2 spike protein. Local alignments of peptides identified in sample B _(A+)_ mapping on three different regions of SARS-CoV-2 reference spike protein (NCBI protein LOCUS YP_009724390.1). Amino acids highlighted in red correspond to changes described in Table 1 and Table 2.

The identified AA changes have been checked for their existence among the observed variations in SARS-CoV-2 sequenced isolates available in GISAID ^16^ at time of writing. As shown in Table 2, all of them except NSP3:A274K in pep51 have been already reported in humans; the majority of them have been never reported in the country of origin of the samples (Italy), the remaining ones have been observed in samples sequenced in Italy, but after the time of collection of the infected sample A (February 2020). Some of the found peptides mapping on the SARS-CoV-2 spike protein are shown in Figure 5.

**Table 2.** Amino acid changes reported in Table 1 have been checked for their existence among the observed variations in SARS-CoV-2 sequenced isolates available GISAID at time of writing. All of them, except one, have been already reported in humans, and only two in Italy. For each amino acid change, the number of occurrences in GISAID isolates is reported, together with details of the first human isolate recorded in GISAID with reported collection date. AA change NSP3:A274K of pep51 has never been reported in human SARS-CoV-2 sequences, but it has been found in beta-CoV genome sequences from bats (isolate hCoV-19/bat/Yunnan/RmYN01/2019, collection date 25-06-2019).

Peptide ID	AA change	Observed in human?	#Occurrence in human	Observed in Italy?	Observed in other than human?	First human isolate recorded in GISAID with reported collection date	Collection date
pep51	NSP3:A274K	No	0	No	Yes	-	-
pep51	NSP3:K280T	Yes	2	No	No	hCoV-19/Finland/HEL-18-471/2021	23/01/2021
pep51	NSP3:V286L	Yes	1	No	Yes	hCoV-19/USA/TX-HMH-MCoV-25096/2021	20/01/2021
pep121	NSP3:L1417I	Yes	4	No	No	hCoV-19/USA/WA-UW163/2020	13/03/2020
pep199	S:D614G	Yes	728,982	Yes	Yes	hCoV-19/Australia/NSW2153/2020	25/01/2020
pep33	S:R634S	Yes	2	No	Yes	hCoV-19/India/MH-NIV-815-3/2020	07/04/2020
pep190	S:S698L	Yes	398	Yes	No	hCoV-19/USA/AZ-TG666166/2020	25/03/2020
pep25	NS3:Y264C	Yes	106	No	No	hCoV-19/Canada/ON-S738/2020	09/04/2020
pep68	N:T205I	Yes	25,665	Yes	No	hCoV-19/Beijing/Wuhan_IME-BJ07/2020	29/01/2020
pep68	N:A208G	Yes	514	No	No	hCoV-19/USA/MD-HP00076/2020	11/03/2020

### Presence of other metabolites

We have already described the detection of toxin-like peptides in plasma, urine and faecal samples from COVID-19 affected individuals (Cristoni *et al.* ^17^, *under review*). The evaluation on the potential release of toxic-like peptides in aliquots from sample B _(A+)_ has been assessed by performing the same analyses. Toxin-like peptides have been observed, but their presence was completely reduced to negligible levels in the aliquots treated with metronidazole and vancomycin administration (data not shown). These results need to be carefully interpreted, taking into account the different antimicrobials kinetics.

### Presence of eukaryotic cells and virus-like particles

**Figure 6.**
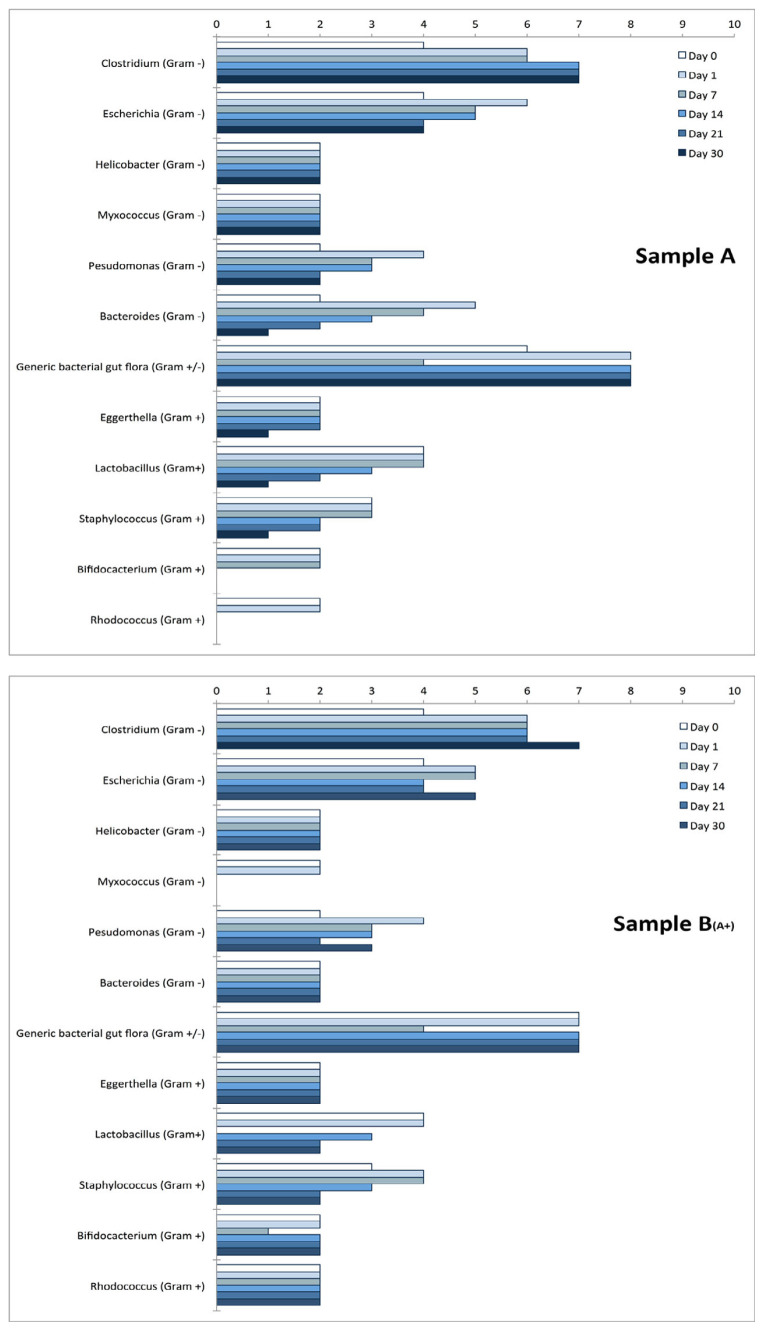
Bacteria genera. The presence of bacteria genera was monitored over time by looking at their metabolic activity as described by Cristoni *et al.* ^14^ Measures on Y-axis are reported as “detection frequency” (range 0–10). The two charts report the most metabolically active genera identified together with the “generic bacterial gut microbiota ” (representing other bacterial genera not classified by the instrument) at day 0, 1, 7, 14, 21, and 30 for samples A, and B _(A+)_. Other microbial organisms were observed at low levels (2 or less, at day 7) and not reported in the figure: *Mycobacterium, Actinobacteria, Bacteroidetes, Blautia, Brevibacterium, Brevundimonas, Candida (C. albicans), Collinsella, Enterococcus, Eubacterium, Klebsiella, Lactonifactor, Microbacterium, Porphyromonas, Propionibacterium, Sphingomonas, Stenotrophomonas, Streptococcus gordonii, Xanthomonas.*

Samples A and B _(A+)_ were found to contain some bacterial genera particularly abundant and metabolically active during the whole experiment, as shown in Figure 6.

Aliquots of samples A, B, and B _(A+),_ collected at different times, were analysed with both transmission electron microscope (TEM) and scanning electron microscope (SEM) to verify the presence of eukaryotic cells. More than 30 different preparations (including at 30 days of culture) have been observed: none was found to contain any structure resembling cells with nuclei, but only bacterial cells. Analyses of images on samples A and B _(A+)_ revealed the presence of virus-like particles interacting with bacterial cells. Immune electron microscopy is ongoing for confirming that these particles are of SARS-CoV-2 origin ( *in preparation*).

## Discussion

Our results indicate that the SARS-CoV-2 genome, in addition to its known interactions with eukaryotic cells, is additionally capable of replicating outside the human body, suggesting a possible ‘bacteriophage-like’ mode of action. It is not clear yet whether the SARS-CoV-2 genome could just be replicated by its RNA polymerase (which would correspond to a bacteriophage pseudo-lysogenic mechanism), or if the production of full-blown SARS-CoV-2 replicating particles within the bacteria occur (which would correspond to the typical lytic cycle of bacteriophages). In either case, according to our knowledge, this is a novel observation and has never described before for SARS-CoV-2.

The experiment here described ( Figure 1) was repeated three additional times using the same samples ( Figure 2). In addition, independent replications were performed in a 3×3 design using different starting material ( Figure 3). In all of them, very similar trends were observed and the increase of SARS-CoV-2 RNA load in sample A and sample B _(A+)_ was confirmed in all experiments.

In the cases of replications with different starting material ( *i.e.* faecal samples from different “infected donors” as sources of A, and “healthy recipients” as sources of B), the trends are similar and confirm that the experiment is reproducible. In terms of relevance, we noticed that SARS-CoV-2 RNA load was particularly high in one combination (A2×B2). It is thus plausible that viral RNA load depends on the gut eubiotic/dysbiotic condition met by the virus. This is also a plausible explanation of why in the initial couple of A-B sources ( Figure 1) the difference between viral RNA load measurements in A and B(A+) is notable. SARS-CoV-2 is considered as a respiratory virus, and many bacteria reside in the upper respiratory tract (URT) interacting with different viruses like influenza (see Schenk *et al*., 2016[MP1] for an overview). With this respect, our observations are in line with the hypothesis formulated by Shah, who [MP2] has recently proposed the existence of a gut-lung equilibrium mediated by multiple mechanisms of action, including the abundance of certain microorganisms in the gut microbiota as responsible for determining the sensitivity and severity of SARS-CoV-2 infections.

Moreover, recent reports suggest an interaction of URT microbiota with SARS-CoV-2 (Ebrahimi, 2020[MP3], Budding *et al*., 2020[MP4]). In particular, Ebrahimi identified *in silico* a series of serine protease TMPRSS2 and peptidyl peptidases with high similarity to the angiotensin-converting enzyme 2 (ACE2) receptor peptidase domain (ACE2-PD) in members of *Proteobacteria* phylum. It can’t be excluded that these or other similar proteins act as the cellular receptors for SARS-CoV-2 in bacteria. At the same time, ACE2 receptor gene, whose protein is known to be critical in SARS-CoV-2 transmission ^18^, is expressed in various tissues and organs of the human body, including the small intestine ^19^. It is thus very likely that SARS-CoV-2 found in faecal samples of infected individuals is from infections occurring in human body cells. Our observations are not in contrast, and they suggest that, in the gastrointestinal tract, human cells, like those of small intestine, are not the sole SARS-CoV-2 target. Looking for taxa, species or consortia that can be prone to act as receptor of virus is imperative, and, with this respect, a whole-genome metagenomic sequencing on the samples is ongoing, aimed at characterising further which bacterial species are candidate target(s) of the observed behaviour of SARS-CoV-2.

Whereas our experimental design was intended to grow bacterial cells, the possibility that SARS-CoV-2 RNA increase could be due to replication in human cells present in the original faecal samples, was considered. The human cells most abundantly present in faecal samples are colonic epithelial cells (colonocytes). Loktionov ^17^, reported that cell exfoliation events from colonic epithelium are rare under normal conditions, while they dramatically increase in cases of uncontrolled growth of cells not under physiologic control (like in neoplasia), when cell removal by apoptosis does not function properly. In addition, Iyengar *et al.* ^20^ reported that colonic epithelial cells terminally differentiated are devoid of proliferative activity. More recently, Nair *et al.* ^21^ and Chandel *et al.* ^22^ developed specific methodologies to recover viable colonocytes from stool. In our case, both sample A and B originated from adult individuals with no diagnosis of cancer. In addition, it is unlikely that human cells potentially present in samples A and B are able to:

- grow in a culture medium typically formulated for bacteria and not containing growth factors, serum, nor other important components for eukaryotic cell sustainment;
- survive in such a medium for 30 days, and in co-occurrence with an event of SARS-CoV-2 infection;
- multiply in the absence of specific CO _2_ concentration conditions.

Also, the possibility of interaction between SARS-CoV-2 and other eukaryotic organisms potentially present in the cultures, as *e.g.* parasitic nematodes and fungal cells, has been considered.

During the whole experiment, parasitic nematodes were not noted at visual inspections by eye. In addition, stool of sample B was independently analysed and certified to be free of known parasites and microbial pathogens (certification provided by the Italian diagnostic laboratory Biomolecular Diagnostic Srl). Parasitic nematodes are usually not able to survive outside the host and many intestinal roundworms (like those of genus *Ascaris*) release antimicrobial factors that interfere with bacterial growth ^23^, in contrast with the found high increase of metabolic activities of some bacterial genera. In the used medium, chemical elements relevant for (parasitic and not) nematodes ( *e.g.* cholesterol and traces of metals) are missing.

The possibility of involvement of the mycobiome fraction present in the stool was considered. As highlighted by Chin *et al.* ^24^, multifaceted and multidisciplinary approaches are necessary to identify uncultivatable, low-abundance, permanent and transient fungal species residing in the gut, confirming that the human mycobiome is not yet fully characterised. Accordingly, while the ability of unknown fungi to grow in the used culture medium cannot be excluded, no significant metabolic activity of *Candida albicans,* most commonly found in the microbiota, was observed.

Finally, inspections of images from TEM and SEM on more than 30 different preparations did not reveal presence of eukaryotic cells ( *in preparation*). If on the one hand, the possibility that a nematode or another unknown eukaryotic cell is able grow in the medium cannot be excluded, the used conditions make this possibility very unlikely. Anyhow, the ability of SARS-CoV-2 to interact either with nematodes or with fungal cells has never been observed before and would be a novel and surprising observation as well.

As indicated above, several peptides matching to SARS-CoV-2 proteins were found in the aliquot from sample B _(A+)_. The identification of peptides with amino acid changes, compared to the translations of CDS regions of the reference SARS-CoV-2 genome, is intriguing but is compatible with the mechanism of viral replication in bacteria. RNA viruses such as SARS-CoV-2 inhabit the host as a population of variants called *quasispecies*, *i.e.* a group made of different variants that are genetically linked through mutation events, and contribute collectively to the characteristics of the whole (viral) population in the host ^25^. Recent studies highlighted the significant amount of intra-host genomic diversity in SARS-CoV-2 samples ^26, 27^. In a ‘bacteriophage-like’ mode of action, as bacteria were grown for 30 days, it can’t be excluded that the observed amino acid changes represent viral *quasispecies* emerged through replication events in bacterial hosts. In relation to this, recent studies ^28– 30^ evidenced hypermutations occurrences in SARS-CoV-2 genomes and suggested APOBEC and ADAR deaminases as the possible responsible of these phenomena. The APOBEC family is related to bacterial, yeast, and plant deaminases all possessing highly conserved amino acid motifs responsible for coordination of zinc in the active site ^31^. As no sequencing was performed on the original infected stool sample, the presence of SARS-CoV-2 haplotypes in the initial SARS-CoV-2 population used to perform the experiments, therefore justifying the amino acid changes observed, cannot be excluded. However, all the amino acid changes found have been reported in sequences of SARS-CoV-2 found for the first time after the date of collection of the infected sample A (February 2020), and some of them have never been reported in Italy, the country of origin of the samples.

On the other hand, other mechanisms like those at the basis of diversity-generating retroelements (DGR) systems ^32^ have that could contribute to SARS-CoV-2 hypermutation phenomena have recently been described in bacteria, and could therefore be responsible of the AA changes found. Additional experiments aimed to verify increase of viral peptides similar to viral RNAover time are planned.

These results can potentially provide new insights in the epidemiology of SARS-CoV-2. Considering the possible impact and implications that such relationship has on the manifestation, therapy and control of COVID-19 disease, some questions immediately arise like *e.g.*:

- Can this ‘bacteriophage-like’ behaviour of SARS-CoV-2 explain the long-term presence of SARS-CoV-2 observed in some recovered patients? ^33^
- Can antibiotics and/or bacteriophage-based therapies play a role in the treatment of COVID-19 affected patients? ^34^
- How would the (antecedent) administration of antibiotics to patients, influencing the microbiota population, impact the clinical course of the disease? ^35^
- Can the involvement of bacteria in COVID-19 epidemiology help to explain clinical observations, like the elevated serum C-reactive protein, procalcitonin, D-dimer, and ferritin associated with poor outcomes in COVID-19? ^36^

These questions are only examples of the plethora of questions to be addressed. Our results support the way to tackle COVID-19 pandemic proposed by Mushi ^37^, *i.e.* by using the One Health holistic approach. If individuals are considered not only human bodies, but as ‘holobionts’, *i.e.* discrete ecological units that need to be studied and treated as such, a deeper understanding of the role of the microbial community living in the human body is fundamental to tackle COVID-19 disease.

## Consent

Faecal samples were collected and handled by CranioMed S.R.L. from anonymous donors who agreed to participate in this study by signing informed consent, as foreseen by Italian legislation. No personal information ( *i.e.* age, sex, blood serotype, severity of the disease, time of the collection, fatality, *etc.*) were collected.

The study is compliant with the JRC Scientific Integrity and Research Ethics guidance.

## Declarations

The scientific output expressed does not imply a policy position of the European Commission. Neither the European Commission nor any person acting on behalf of the Commission is responsible for the use that might be made of this publication.

## Data availability

### Underlying data

Zenodo: Underlying data for ‘Increase of SARS-CoV-2 RNA load in faecal samples prompts for rethinking of SARS-CoV-2 biology and COVID-19 epidemiology’. https://doi.org/10.5281/zenodo.4723549 ^15^.

The project contains the following underlying data:

Mass spectrometry raw data of the peptides.

NCBI Genome: Severe acute respiratory syndrome coronavirus 2 isolate Wuhan-Hu-1, complete genome. Accession number: NC_045512.2; https://www.ncbi.nlm.nih.gov/nuccore/NC_045512.2.

NCBI Protein: Surface glycoprotein [Severe acute respiratory syndrome coronavirus 2]. Accession number: YP_009724390.1; https://www.ncbi.nlm.nih.gov/protein/1796318598.

### Extended data

Zenodo: Extended data for ‘Increase of SARS-CoV-2 RNA load in faecal samples prompts for rethinking of SARS-CoV-2 biology and COVID-19 epidemiology’. https://doi.org/10.5281/zenodo.4723549 ^15^.

Data are available under the terms of the Creative Commons Attribution 4.0 International license (CC-BY 4.0).

## Authors’ notes

The efforts and commitment put into this research work are dedicated to all EU citizens who suddenly left us because of COVID-19, and to their families.
