# Supplementary material for: Increase of SARS-CoV-2 RNA load in faecal samples prompts for rethinking of SARS-CoV-2 biology and COVID-19 epidemiology
Source: F1000Res. 2021 Jul 1;10:370. Originally published 2021 May 11. [Version 3] doi: 10.12688/f1000research.52540.3 (PMC8283343; doi:10.12688/f1000research.52540.3)
Supplement: Peer review history

# Peer review: Reviewer response for version 3

**DOI:** 10.5256/f1000research.58157.r88861

**Published:** 2021-07-01

**Authors:** Kourosh Honarmand Ebrahimi

**Affiliations:** 1 Department of Chemistry, University of Oxford, Oxford, UK

I thank the authors for all their efforts. They have fully answered my concerns. I recommend the indexing of this work.

Is the work clearly and accurately presented and does it cite the current literature?

Partly

If applicable, is the statistical analysis and its interpretation appropriate?

Yes

Are all the source data underlying the results available to ensure full reproducibility?

Yes

Is the study design appropriate and is the work technically sound?

Yes

Are the conclusions drawn adequately supported by the results?

Partly

Are sufficient details of methods and analysis provided to allow replication by others?

Partly

Reviewer Expertise:

Virology and immunology and bioinorganic chemistry

I confirm that I have read this submission and believe that I have an appropriate level of expertise to confirm that it is of an acceptable scientific standard.

# Peer review: Reviewer response for version 2

**DOI:** 10.5256/f1000research.57680.r88114

**Published:** 2021-06-25

**Authors:** Kourosh Honarmand Ebrahimi

**Affiliations:** 1 Department of Chemistry, University of Oxford, Oxford, UK

The authors have fully addressed my original concerns. I found their hypothesis very interesting, however, they should note and somewhere in the paper they mention that ACE2 receptor is expressed in other tissues like the small intestine, and what they have observed could be due to the replication of the virus in the gastrointestinal tract tissue (Han *et al.* (2020 ^1^)).

Is the work clearly and accurately presented and does it cite the current literature?

Partly

If applicable, is the statistical analysis and its interpretation appropriate?

Yes

Are all the source data underlying the results available to ensure full reproducibility?

Yes

Is the study design appropriate and is the work technically sound?

Yes

Are the conclusions drawn adequately supported by the results?

Partly

Are sufficient details of methods and analysis provided to allow replication by others?

Partly

Reviewer Expertise:

Virology and immunology and bioinorganic chemistry

I confirm that I have read this submission and believe that I have an appropriate level of expertise to confirm that it is of an acceptable scientific standard, however I have significant reservations, as outlined above.

## References

[1] : Analysis of 2019-nCoV receptor ACE2 expression in different tissues and its significance study. *Ann Transl Med* .2020;8(17) : 10.21037/atm-20-4281 1077 10.21037/atm-20-4281 33145296PMC7576005

# Response

**Published:** 2021-06-25

**Authors:** Mauro Petrillo

**Affiliations:** European Commission, Joint Research Centre (JRC), Ispra, Italy

Dear Dr Kourosh Honarmand Ebrahimi,

 Thanks a lot for your comment.

 Your point is relevant and we modified the Discussion section accordingly:

*Moreover, recent reports suggest an interaction of URT microbiota with SARS-CoV-2 (Ebrahimi, 2020, Budding et al., 2020). In particular, Ebrahimi identified in silico a series of serine protease TMPRSS2 and peptidyl peptidases with high similarity to the angiotensin-converting enzyme 2 (ACE2) receptor peptidase domain (ACE2-PD) in members of Proteobacteria phylum. It can’t be excluded that these or other similar proteins act as the cellular receptors for SARS-CoV-2 in bacteria. **At the same time, ACE2 receptor gene, whose protein is known to be critical in SARS-CoV-2 transmission [Yan 2020], is expressed in various tissues and organs of the human body, including the small intestine [Han 2020]. It is thus very likely that SARS-CoV-2 found in faecal samples of infected individuals is from infections occurring in human body cells. Our observations are not in contrast, and they suggest that, in the gastrointestinal tract, human cells, like those of small intestine, are not the sole SARS-CoV-2 target.***

 We hope that the quality of the manuscript, thanks to your comment, has been improved and you consider it suitable for publication.

 Best regards,

 Mauro Petrillo, on behalf of the authors.

# Peer review: Reviewer response for version 2

**DOI:** 10.5256/f1000research.57680.r88113

**Published:** 2021-06-25

**Authors:** Margarita Aguilera

**Affiliations:** 1 Department of Microbiology, Faculty of Pharmacy, Campus of Cartuja, University of Granada, Granada, Spain

Authors have fully improved the manuscript according to all the suggestions proposed. No further actions are needed.

Is the work clearly and accurately presented and does it cite the current literature?

Yes

If applicable, is the statistical analysis and its interpretation appropriate?

Yes

Are all the source data underlying the results available to ensure full reproducibility?

Partly

Is the study design appropriate and is the work technically sound?

Yes

Are the conclusions drawn adequately supported by the results?

Yes

Are sufficient details of methods and analysis provided to allow replication by others?

Partly

Reviewer Expertise:

Microbiota, Probiotics, taxonomy, Microbiology, Molecular biology, Biotechnology.

I confirm that I have read this submission and believe that I have an appropriate level of expertise to confirm that it is of an acceptable scientific standard.

# Response

**Published:** 2021-06-25

**Authors:** Mauro Petrillo

**Affiliations:** European Commission, Joint Research Centre (JRC), Ispra, Italy

Dear Prof. Aguilera,

 Thanks a lot for your valuable comments and suggestions that you provided. They surely improved the quality of the manuscript.

 Best regards,

 Mauro Petrillo, on behalf of the authors.

# Peer review: Reviewer response for version 1

**DOI:** 10.5256/f1000research.55836.r85802

**Published:** 2021-06-07

**Authors:** Margarita Aguilera

**Affiliations:** 1 Department of Microbiology, Faculty of Pharmacy, Campus of Cartuja, University of Granada, Granada, Spain

The work titled “ *Increase of SARS-CoV-2 RNA load in faecal samples prompts for rethinking of SARS-CoV-2 biology and COVID-19 epidemiology” *has been well designed and soundly performed.

 After the urgent needs to invest efforts on clinical research for handling the COVID-19, new questions should be posed and the present work has done innovatively. The hypothesis is relevant due to the high differential effects within SARS-CoV-2 patients, based on the individual health statuses that determine variable host responses. The Authors have shown experimental data to demonstrate that SARS-CoV-2 could have a bacteriophage activity through evaluating SARS-CoV-2 long-term survival, proliferation and the potential interaction between the virus and gut microbiota taxa. Multiple methodologies and experimental data support appropriately the results and conclusions: viral RNA by Luminex technology; impact of antibiotic treatments; Biotyper for cultured microbiota predominant taxa over the time, eukaryotic cells, peptides, other metabolites molecular alignments, etc.

 Therefore, I consider that Petrillo *et al.* have carried out an excellent and holistic approach; **I would suggest its publication after minor corrections/explanations:**

- Please use the term gut microbiota instead gut flora along the document.

- Please add the underlined phrase at the end of this paragraph: *At the same time, Wölfel et al.12 observed high viral RNA concentration in stool samples, but reported isolation of infectious virus only from throat- and lung-derived samples, while Yao et al. had indication of viable SARS-CoV-2 particles in stool samples, denoting that the detailed biology of SARS-CoV-2 is not yet fully elucidated.* Moreover, the interaction between SARS-CoV-2 and individual variable microbiota composition could drive the differential pathophysiological effects and severity of symptoms.
- Please explain if the medium NutriSelect™Plus nutrient used for the inoculation and cultivation of microorganisms could have an impact on the specific taxa cultured as well as metabolites measured. Have you tried other media?
- Please explain if you have been used only aerobic conditions for all experimental culturing data. 
- Please specify the concentration of antibiotics added to the experiment.
- Figure 1: Please give a plausible explanation for the data shown in this figure - Could it be that microbiota of healthy individuals contain eubiotic taxa that allow a better proliferation of SARS-CoV-2?
- Figure 3: Please explain the differences observed between the three graphics. The microbiota composition of the sample receptors seems to have an impact. Please give a plausible explanation.

- Future research is directly derived from these results: to look for the taxa, species or consortia that can be prone to act as receptor of virus. Please comment on that.  

Is the work clearly and accurately presented and does it cite the current literature?

Yes

If applicable, is the statistical analysis and its interpretation appropriate?

Yes

Are all the source data underlying the results available to ensure full reproducibility?

Partly

Is the study design appropriate and is the work technically sound?

Yes

Are the conclusions drawn adequately supported by the results?

Yes

Are sufficient details of methods and analysis provided to allow replication by others?

Partly

Reviewer Expertise:

Microbiota, Probiotics, taxonomy, Microbiology, Molecular biology, Biotechnology.

I confirm that I have read this submission and believe that I have an appropriate level of expertise to confirm that it is of an acceptable scientific standard.

# Response

**Published:** 2021-06-07

**Authors:** Mauro Petrillo

**Affiliations:** European Commission, Joint Research Centre (JRC), Ispra, Italy

Dear Prof. Aguilera,

 Thanks a lot for your valuable comments and suggestions that you have provided in the report.

 We will address all of them, and will provide a revised version of the manuscript.

 Best regards,

 Mauro Petrillo, on behalf of the authors.

# Response

**Published:** 2021-06-09

**Authors:** Mauro Petrillo

**Affiliations:** European Commission, Joint Research Centre (JRC), Ispra, Italy

Dear Dr Aguilera,

 Thanks a lot for your valuable comments and suggestions that you have provided in the report.

 As anticipated, we have addressed all your points, and provided a new version of the manuscript:

  

- *Please use the term gut microbiota instead gut flora along the document.*
- **Response: **Done, thanks.
- *Please add the underlined phrase at the end of this paragraph: 'At the same time, Wölfel et al.12 observed high viral RNA concentration in stool samples, but reported isolation of infectious virus only from throat- and lung-derived samples, while Yao et al. had indication of viable SARS-CoV-2 particles in stool samples, denoting that the detailed biology of SARS-CoV-2 is not yet fully elucidated. Moreover, the interaction between SARS-CoV-2 and individual variable microbiota composition could drive the differential pathophysiological effects and severity of symptoms.'*
- **Response: **Very relevant point. We added it, together with references.
- *Please explain if the medium NutriSelect™Plus nutrient used for the inoculation and cultivation of microorganisms could have an impact on the specific taxa cultured as well as metabolites measured. Have you tried other media?*
- *Please explain if you have been used only aerobic conditions for all experimental culturing data. *
- *Please specify the concentration of antibiotics added to the experiment.*
- **Response: **With respect to these points, the information has been added in the supplementary material, points 1, 3, 8). The new link for get the supplementary material is https://doi.org/10.5281/zenodo.4723549

 

- *Figure 1: Please give a plausible explanation for the data shown in this figure - Could it be that microbiota of healthy individuals contain eubiotic taxa that allow a better proliferation of SARS-CoV-2?*
- *Figure 3: Please explain the differences observed between the three graphics. The microbiota composition of the sample receptors seems to have an impact. Please give a plausible explanation.*
- *Future research is directly derived from these results: to look for the taxa, species or consortia that can be prone to act as receptor of virus. Please comment on that.*
- **Response: **Your points are relevant and we modified the Discussion section accordingly: 'In terms of relevance, we noticed that SARS-CoV-2 RNA load was particularly high in one combination (A2×B2). It is thus plausible that viral RNA load depends on the gut eubiotic/dysbiotic condition met by the virus. This is also a plausible explanation of why in the initial couple of A-B sources (Figure 1) the difference between viral RNA load measurements in A and B(A+) is notable.SARS-CoV-2 is considered as a respiratory virus, and many bacteria reside in the upper respiratory tract (URT) interacting with different viruses like influenza (see Schenk et al., 2016 for an overview). With this respect, our observations are in line with the hypothesis formulated by Shah, who has recently proposed the existence of a gut-lung equilibrium mediated by multiple mechanisms of action, including the abundance of certain microorganisms in the gut microbiota as responsible for determining the sensitivity and severity of SARS-CoV-2 infections. Moreover, recent reports suggest an interaction of URT microbiota with SARS-CoV-2 (Ebrahimi, 2020, Budding et al., 2020). In particular, Ebrahimi identified in silico a series of serine protease TMPRSS2 and peptidyl peptidases with high similarity to the ACE2 peptidase domain (ACE2-PD) in members of Proteobacteria phylum. It can’t be excluded that these or other similar proteins act as the cellular receptors for SARS-CoV-2 in bacteria. Looking for taxa, species or consortia that can be prone to act as receptor of virus is imperative, and, with this respect, a whole-genome metagenomic sequencing on the samples is ongoing, aimed at characterising further which bacterial species are candidate target(s) of the observed behaviour of SARS-CoV-2.'

 

 We believe that, thanks to your comments, the quality of the manuscript has improved a lot.

 Best regards,

 Mauro Petrillo, on behalf of the authors.

# Peer review: Reviewer response for version 1

**DOI:** 10.5256/f1000research.55836.r85393

**Published:** 2021-05-17

**Authors:** Kourosh Honarmand Ebrahimi

**Affiliations:** 1 Department of Chemistry, University of Oxford, Oxford, UK

Petrillo and colleagues report that SARS-CoV-2 could have a bacteriophage activity in faecal samples. They measure the presence of viral RNA using Luminex technology and viral proteins using mass spectrometry. The experimental design is sound and their findings are exciting, which support the publication of this work. However, I have few concerns that must be addressed before publication. Therefore, I recommend publication after minor revisions.

- SARS-CoV-2 is a respiratory virus. Many bacteria reside in the upper respiratory tract (URT) and interact with different viruses like influenza (Schenk *et al.*, 2016 ^1^). Moreover, ****recent reports suggest an interaction of URT microbiota with SARS-CoV-2  (e.g. (Ebrahimi, 2020 ^2^); (Budding *et al.*, 2020 ^3^)). The authors should cite these literatures and discuss their findings with respect to a similar bacteriophage behaviour of SARS-CoV-2 in URT.
- In Figure 3, the graph for donor A2: Why in B2(A2+) sample the amount of RNA is hugely different than the other samples? The authors need to explain.
- The authors suggest that the presence of SARS-CoV-2 peptides is compatible with the mechanism of viral replication in bacteria. If this is true, shouldn't the authors observe an increase in viral peptides similar to viral RNA?

Is the work clearly and accurately presented and does it cite the current literature?

Partly

If applicable, is the statistical analysis and its interpretation appropriate?

Yes

Are all the source data underlying the results available to ensure full reproducibility?

Yes

Is the study design appropriate and is the work technically sound?

Yes

Are the conclusions drawn adequately supported by the results?

Partly

Are sufficient details of methods and analysis provided to allow replication by others?

Partly

Reviewer Expertise:

Virology and immunology

I confirm that I have read this submission and believe that I have an appropriate level of expertise to confirm that it is of an acceptable scientific standard, however I have significant reservations, as outlined above.

## References

[1] : Composition and immunological significance of the upper respiratory tract microbiota. *FEBS Lett* .2016;590(21) : 10.1002/1873-3468.12455 3705-3720 10.1002/1873-3468.12455 27730630PMC7164007

[2] : SARS-CoV-2 spike glycoprotein-binding proteins expressed by upper respiratory tract bacteria may prevent severe viral infection. *FEBS Lett* .594(11) : 10.1002/1873-3468.13845 1651-1660 10.1002/1873-3468.13845 32449939PMC7280584

[3] : An Age Dependent Pharyngeal Microbiota Signature Associated with SARS-CoV-2 Infection. *SSRN Electronic Journal* .2020; 10.2139/ssrn.3582780 10.2139/ssrn.3582780

# Response

**Published:** 2021-05-17

**Authors:** Mauro Petrillo

**Affiliations:** European Commission, Joint Research Centre (JRC), Ispra, Italy

Dear Dr Kourosh Honarmand Ebrahimi,

 Thanks a lot for your valuable comments and suggestions that you have provided in the report.

 We will address all of them, and wait for those of other reviewers, in order to provide a fully revised version of the manuscript.

 Best regards,

 Mauro Petrillo, on behalf of the authors.

# Response

**Published:** 2021-06-09

**Authors:** Mauro Petrillo

**Affiliations:** European Commission, Joint Research Centre (JRC), Ispra, Italy

Dear Dr Kourosh Honarmand Ebrahimi,

 Thanks a lot for your valuable comments and suggestions that you have provided in the report.

 As anticipated, we have addressed all your points, and provided a new version of the manuscript:

  

- *SARS-CoV-2 is a respiratory virus. Many bacteria reside in the upper respiratory tract (URT) and interact with different viruses like influenza (Schenk et al., 2016). Moreover, ****recent reports suggest an interaction of URT microbiota with SARS-CoV-2  (e.g. (Ebrahimi, 2020); (Budding et al., 2020). The authors should cite these literatures and discuss their findings with respect to a similar bacteriophage behaviour of SARS-CoV-2 in URT.*
- **Response: **Your point is relevant and we modified the Discussion section accordingly: 'SARS-CoV-2 is considered as a respiratory virus, and many bacteria reside in the upper respiratory tract (URT) interacting with different viruses like influenza (see Schenk *et al*., 2016 for an overview). With this respect, our observations are in line with the hypothesis formulated by Shah, who has recently proposed the existence of a gut-lung equilibrium mediated by multiple mechanisms of action, including the abundance of certain microorganisms in the gut microbiota as responsible for determining the sensitivity and severity of SARS-CoV-2 infections. Moreover, recent reports suggest an interaction of URT microbiota with SARS-CoV-2 (Ebrahimi, 2020, Budding *et al.*, 2020). In particular, Ebrahimi identified in silico a series of serine protease TMPRSS2 and peptidyl peptidases with high similarity to the ACE2 peptidase domain (ACE2-PD) in members of Proteobacteria phylum. It can’t be excluded that these or other similar proteins act as the cellular receptors for SARS-CoV-2 in bacteria. Looking for taxa, species or consortia that can be prone to act as receptor of virus is imperative, and, with this respect, a whole-genome metagenomic sequencing on the samples is ongoing, aimed at characterising further which bacterial species are candidate target(s) of the observed behaviour of SARS-CoV-2.'

 

- *In Figure 3, the graph for donor A2: Why in B2(A2+) sample the amount of RNA is hugely different than the other samples? The authors need to explain.*
- **Response: **We have modified Figure 3, by adding an additional panel. In addition, we modified the Discussion section accordingly: 'In terms of relevance, we noticed that SARS-CoV-2 RNA load was particularly high in one combination (A2×B2). It is thus plausible that viral RNA load depends on the gut eubiotic/dysbiotic condition met by the virus. This is also a plausible explanation of why in the initial couple of A-B sources (Figure 1) the difference between viral RNA load measurements in A and B(A+) is notable.'
* *
- *The authors suggest that the presence of SARS-CoV-2 peptides is compatible with the mechanism of viral replication in bacteria. If this is true, shouldn't the authors observe an increase in viral peptides similar to viral RNA?*
- **Response: **Your point is relevant and we modified the Discussion section accordingly: 'Additional experiments aimed to verify increase of viral peptides similar to viral RNA over time are planned.'

We hope that the quality of the manuscript, thanks to your comments, has been improved and you consider it suitable for publication.

 Best regards,

 Mauro Petrillo, on behalf of the authors.
