# Supplementary material for: Increase of SARS-CoV-2 RNA load in faecal samples prompts for rethinking of SARS-CoV-2 biology and COVID-19 epidemiology
Source: F1000Res. 2021 May 11;10:370. [Version 1] doi: 10.12688/f1000research.52540.1 (PMC8283343; doi:10.12688/f1000research.52540.1)
Supplement: Peer review history

# Response

**Published:** 2021-06-09

**Authors:** Mauro Petrillo

**Affiliations:** European Commission, Joint Research Centre (JRC), Ispra, Italy

Dear Dr Aguilera,

 Thanks a lot for your valuable comments and suggestions that you have provided in the report.

 As anticipated, we have addressed all your points, and provided a new version of the manuscript:

- *Please use the term gut microbiota instead gut flora along the document.*
- **Response: **Done, thanks.
- *Please add the underlined phrase at the end of this paragraph: 'At the same time, Wölfel et al.12 observed high viral RNA concentration in stool samples, but reported isolation of infectious virus only from throat- and lung-derived samples, while Yao et al. had indication of viable SARS-CoV-2 particles in stool samples, denoting that the detailed biology of SARS-CoV-2 is not yet fully elucidated. Moreover, the interaction between SARS-CoV-2 and individual variable microbiota composition could drive the differential pathophysiological effects and severity of symptoms.'*
- **Response: **Very relevant point. We added it, together with references.
- *Please explain if the medium NutriSelect™Plus nutrient used for the inoculation and cultivation of microorganisms could have an impact on the specific taxa cultured as well as metabolites measured. Have you tried other media?*
- *Please explain if you have been used only aerobic conditions for all experimental culturing data. *
- *Please specify the concentration of antibiotics added to the experiment.*
- **Response: **With respect to these points, the information has been added in the supplementary material, points 1, 3, 8). The new link for get the supplementary material is https://doi.org/10.5281/zenodo.4723549

- *Figure 1: Please give a plausible explanation for the data shown in this figure - Could it be that microbiota of healthy individuals contain eubiotic taxa that allow a better proliferation of SARS-CoV-2?*
- *Figure 3: Please explain the differences observed between the three graphics. The microbiota composition of the sample receptors seems to have an impact. Please give a plausible explanation.*
- *Future research is directly derived from these results: to look for the taxa, species or consortia that can be prone to act as receptor of virus. Please comment on that.*
- **Response: **Your points are relevant and we modified the Discussion section accordingly: 'In terms of relevance, we noticed that SARS-CoV-2 RNA load was particularly high in one combination (A2×B2). It is thus plausible that viral RNA load depends on the gut eubiotic/dysbiotic condition met by the virus. This is also a plausible explanation of why in the initial couple of A-B sources (Figure 1) the difference between viral RNA load measurements in A and B(A+) is notable.SARS-CoV-2 is considered as a respiratory virus, and many bacteria reside in the upper respiratory tract (URT) interacting with different viruses like influenza (see Schenk et al., 2016 for an overview). With this respect, our observations are in line with the hypothesis formulated by Shah, who has recently proposed the existence of a gut-lung equilibrium mediated by multiple mechanisms of action, including the abundance of certain microorganisms in the gut microbiota as responsible for determining the sensitivity and severity of SARS-CoV-2 infections. Moreover, recent reports suggest an interaction of URT microbiota with SARS-CoV-2 (Ebrahimi, 2020, Budding et al., 2020). In particular, Ebrahimi identified in silico a series of serine protease TMPRSS2 and peptidyl peptidases with high similarity to the ACE2 peptidase domain (ACE2-PD) in members of Proteobacteria phylum. It can’t be excluded that these or other similar proteins act as the cellular receptors for SARS-CoV-2 in bacteria. Looking for taxa, species or consortia that can be prone to act as receptor of virus is imperative, and, with this respect, a whole-genome metagenomic sequencing on the samples is ongoing, aimed at characterising further which bacterial species are candidate target(s) of the observed behaviour of SARS-CoV-2.'

We believe that, thanks to your comments, the quality of the manuscript has improved a lot.

 Best regards,

 Mauro Petrillo, on behalf of the authors.

# Response

**Published:** 2021-06-09

**Authors:** Mauro Petrillo

**Affiliations:** European Commission, Joint Research Centre (JRC), Ispra, Italy

Dear Dr Kourosh Honarmand Ebrahimi,

 Thanks a lot for your valuable comments and suggestions that you have provided in the report.

 As anticipated, we have addressed all your points, and provided a new version of the manuscript:

- *SARS-CoV-2 is a respiratory virus. Many bacteria reside in the upper respiratory tract (URT) and interact with different viruses like influenza (Schenk et al., 2016). Moreover, ****recent reports suggest an interaction of URT microbiota with SARS-CoV-2  (e.g. (Ebrahimi, 2020); (Budding et al., 2020). The authors should cite these literatures and discuss their findings with respect to a similar bacteriophage behaviour of SARS-CoV-2 in URT.*
- **Response: **Your point is relevant and we modified the Discussion section accordingly: 'SARS-CoV-2 is considered as a respiratory virus, and many bacteria reside in the upper respiratory tract (URT) interacting with different viruses like influenza (see Schenk *et al*., 2016 for an overview). With this respect, our observations are in line with the hypothesis formulated by Shah, who has recently proposed the existence of a gut-lung equilibrium mediated by multiple mechanisms of action, including the abundance of certain microorganisms in the gut microbiota as responsible for determining the sensitivity and severity of SARS-CoV-2 infections. Moreover, recent reports suggest an interaction of URT microbiota with SARS-CoV-2 (Ebrahimi, 2020, Budding *et al.*, 2020). In particular, Ebrahimi identified in silico a series of serine protease TMPRSS2 and peptidyl peptidases with high similarity to the ACE2 peptidase domain (ACE2-PD) in members of Proteobacteria phylum. It can’t be excluded that these or other similar proteins act as the cellular receptors for SARS-CoV-2 in bacteria. Looking for taxa, species or consortia that can be prone to act as receptor of virus is imperative, and, with this respect, a whole-genome metagenomic sequencing on the samples is ongoing, aimed at characterising further which bacterial species are candidate target(s) of the observed behaviour of SARS-CoV-2.'

- *In Figure 3, the graph for donor A2: Why in B2(A2+) sample the amount of RNA is hugely different than the other samples? The authors need to explain.*
- **Response: **We have modified Figure 3, by adding an additional panel. In addition, we modified the Discussion section accordingly: 'In terms of relevance, we noticed that SARS-CoV-2 RNA load was particularly high in one combination (A2×B2). It is thus plausible that viral RNA load depends on the gut eubiotic/dysbiotic condition met by the virus. This is also a plausible explanation of why in the initial couple of A-B sources (Figure 1) the difference between viral RNA load measurements in A and B(A+) is notable.'
* *
- *The authors suggest that the presence of SARS-CoV-2 peptides is compatible with the mechanism of viral replication in bacteria. If this is true, shouldn't the authors observe an increase in viral peptides similar to viral RNA?*
- **Response: **Your point is relevant and we modified the Discussion section accordingly: 'Additional experiments aimed to verify increase of viral peptides similar to viral RNA over time are planned.'

We hope that the quality of the manuscript, thanks to your comments, has been improved and you consider it suitable for publication.

 Best regards,

 Mauro Petrillo, on behalf of the authors.
